# Platelet response following dexamethasone in obese vs nonobese patients with primary, acute immune-mediated thrombocytopenia

**DOI:** 10.1016/j.rpth.2025.102844

**Published:** 2025-03-30

**Authors:** Tyler Everhardt, Kelley Julian, Russell Benefield, Aaron Wilson, Nathan Wilson, Charles J. Parker, Anna Parks, Jeffrey A. Gilreath

**Affiliations:** 1 Department of Pharmacy, Huntsman Cancer Institute, University of Utah Health, Salt Lake City, Utah, USA; 2 Department of Pharmacy, University of Utah Health, Salt Lake City, Utah, USA; 3 College of Pharmacy, University of Utah, Salt Lake City, Utah, USA; 4 Division of Hematology, School of Medicine, University of Utah, Salt Lake City, Utah, USA

**Keywords:** corticosteroids, dexamethasone, glucocorticoids, idiopathic, immune thrombocytopenia, obese, obesity, purpura, thrombocytopenic

## Abstract

**Background:**

Immune thrombocytopenia (ITP) is a rare autoimmune disorder defined as a platelet count <100,000/μL, where secondary causes of thrombocytopenia have been excluded. Glucocorticoids are firstline therapy for ITP; however, data and recommendations on the impact of body weight and repeat steroid courses remain limited.

**Objectives:**

We aimed to evaluate if body weight altered the response rates to dexamethasone (DEX) in the treatment of ITP.

**Methods:**

We conducted a retrospective review to evaluate the effects of body weight on response to DEX in ITP. Patients were compared based on body mass index, presentation of ITP (acute or chronic), and cause of ITP (primary or secondary). Initial response, complete response, and relapse rates were among the outcomes investigated among the primary acute ITP population.

**Results:**

Overall, 117 patients with ITP were identified, 49 of whom had primary acute ITP. Of these, 28 were categorized as nonobese, while 21 were obese. Nonobese patients were more likely to experience an initial platelet response to DEX than obese patients (93% vs 71%; *P* = .04), with 68% of nonobese patients also demonstrating a complete response compared with 48% of obese patients. Among patients who did not respond after 1 course of DEX, only 2 patients received another course prior to the initiation of alternative therapies. This is the second study to show that obese patients with primary acute ITP have significantly lower initial response rates and lower complete response rates to DEX compared with nonobese patients and that repeat DEX courses may be underutilized across all body mass index subgroups.

**Conclusion:**

This study further highlights the need for additional data and guidance on optimal glucocorticoid dosing, especially in patients with obesity.

## Essentials

• The impact of body weight on dexamethasone response for ITP is unknown.
• ITP patients treated with dexamethasone were reviewed to evaluate the effects of body weight.
• Obese patients were less likely to have an initial platelet response than nonobese patients.
• Repeat courses of glucocorticoids in the treatment of ITP remain underutilized.

While several new treatment options for chronic immune thrombocytopenia (ITP) have become available over the past 20 years, glucocorticoids remain the preferred front-line therapy for acute ITP [1]. The current American Society of Hematology guideline recommends either a course of oral dexamethasone (DEX) at a fixed dose of 40 mg daily for 4 consecutive days or prednisone 0.5 to 2 mg/kg/d tapered over less than 6 weeks [2]. For refractory disease, however, recommendations for subsequent courses of DEX are lacking. Consequently, clinicians often forgo repeat DEX after suboptimal initial response or during relapse. To date, 11 publications [[3], [4], [5], [6], [7], [8], [9], [10], [11], [12], [13]] have described the effective use of repeat DEX courses, which collectively demonstrate overall response rates ranging from 63% to 100%.

While DEX is generally considered safe and effective, data are limited for predicting response or assessing need for subsequent courses using patient-level characteristics. As with the general population, obesity is common among patients with ITP and may contribute to less effective ITP responses through mechanisms, including altered metabolism of corticosteroids and immune dysregulation through augmentation of function of T cells, Tregs, or myeloid-derived suppressor cells [14,15]. Evidence of the effects of obesity on response to DEX treatment is needed. Herein, we aimed to determine whether response rates to DEX differed between nonobese and obese patients with primary acute ITP and if a higher percentage of obese patients require repeated courses of DEX to achieve a response.

To evaluate platelet responses in obese and nonobese patients after DEX 40 mg × 4 days, a single-center retrospective chart review approved by the University of Utah Institutional Review Board was performed that included data from May 2016 to July 2022. Eligible patients were 18 years or older, diagnosed with ITP, and had a minimum of 2 platelet counts documented within 30 days of their initial DEX dose. Patients receiving concomitant intravenous immunoglobulin (IVIG) were included. Obesity was defined based on calculated body mass index (BMI), with patients categorized as obese (BMI ≥ 30 mg/m^2^) or nonobese (BMI < 30 mg/m^2^). Outcomes were assessed over 6 months. The primary outcome was initial overall response rate, defined as an increase in platelet count to ≥30,000/μL with ≥2-fold increase from baseline after DEX therapy, assessed within 30 days of the first dose of DEX [16]. Additional outcomes included the following: complete (platelet count ≥ 100 × 10^9^/L and absence of bleeding), partial (platelet count ≥30,000/μL and <100,000/μL with ≥2-fold increase from baseline and absence of bleeding), and no response; DEX courses needed to achieve a response; DEX treatment courses within 6 months; time to response; IVIG administration; platelet transfusion; requirement for alternative therapy, including thrombopoietin receptor agonists, rituximab, or splenectomy within 6 months of initial presentation; bleeding following DEX treatment; relapse within 6 months. For univariate comparisons between obese and nonobese patients, differences were assessed for statistical significance using the chi-square test and Wilcoxon rank sum test, as appropriate.

A total of 614 patients were identified using the institutional electronic medical record, thrombocytopenia diagnosis codes, and DEX prescriptions or administrations. Of those, 497 were excluded because thrombocytopenia was not solely attributable to ITP, resulting in 117 eligible patients: 71 nonobese and 46 obese. Of these, 49 were categorized as having primary (no underlying disease associated with ITP), acute (<3 months from diagnosis) ITP (Table 1). Twenty-one (43%) were obese, which is notably similar to the national obesity rate of 42% [17]. Baseline characteristics were comparable between cohorts with the exception of sex assigned at birth, where 71% of obese patients were male compared with 46% nonobese patients. The median BMI was 33 kg/m^2^ vs 24 kg/m^2^ in obese vs nonobeses patients, resulting in median DEX dose of 1.55 mg/kg/course in obese patients compared with a median dose of 2.32 mg/kg/course in nonobese patients (*P* < .0001).

**Table 1:** Demographics of patients with primary, acute immune thrombocytopenia.

Patient characteristics	Total (*N* = 49)	Obese (*n* = 21)	Nonobese (*n* = 28)
Age (y), median (IQR)	55 (34-68)	53 (34-66)	56 (34-74)
Sex (male), *n* (%)	28 (57)	15 (71)	13 (46)
Weight (kg), median (IQR)	89 (67-103)	103 (97-124)	69 (61-79)
BMI (kg/m^2^), median (IQR)	29 (24-33)	33 (32-41)	24 (22-28)
Race, White, *n* (%)	37 (76)	16 (76)	21 (75)
Platelet count on presentation (×10^9^/L), median (range)	6 (5-38)	5 (5-32)	9 (5-27)
Location of initial treatment, inpatient, *n* (%)	32 (65)	13 (62)	19 (68)

BMI, body mass index.

For the primary outcome, a significantly higher proportion of nonobese patients with primary acute ITP patients achieved an initial response to DEX compared with the obese group (93% vs 71%; *P* = .04; Table 2). Additionally, 68% of nonobese patients achieved a complete response compared with 48% of obese patients. There was a nonstatistically significant trend in the number of DEX courses needed to achieve a response between obese and nonobese patients (*P* = .06; Table 2). A higher proportion of nonobese patients achieved 6 months of relapse-free survival; however, this did not reach statistical significance (*P* = .36; Figure).

**Table 2:** Outcomes of patients with primary, acute immune thrombocytopenia.

Primary, acute ITP population	Total (*N* = 49)	Obese (*n* = 21)	Nonobese (*n* = 28)	*P* value
Initial response, *n* (%)^a^	41 (84)	15 (71)	26 (93)	.04
Response type, *n* (%)^b^				.12
Complete response	29 (59)	10 (48)	19 (68)	
Partial response	12 (25)	5 (24)	7 (25)	
No response	8 (16)	6 (28)	2 (7)	
Dexamethasone courses needed to achieve response, median (range)^c^	1 (1-3)	1 (1-3)	1 (1)	.06
No. of dexamethasone treatment courses in 6 mo, median (range)	1 (1-4)	2 (1-4)	1 (1-4)	.71
Time to response (d), median (range)^c^	2 (1-24)	3 (1-24)	2 (1-8)	.65
IVIG administration, *n* (%)	24 (49)	13 (62)	11 (39)	.12
Requirement for platelet infusions, *n* (%)	11 (22)	5 (24)	6 (21)	.84
Alternative therapy following dexamethasone ± IVIG, *n* (%)^d^	30 (61)	15 (71)	15 (54)	.20
Relapse within 6 mo^c^^,^^e^	20 (51)	9 (60)	11 (42)	.27
Bleeding during treatment, *n* (%)	19 (39)	10 (48)	9 (32)	.27

The *a priori* alpha for statistical comparisons was set at .05.

ITP, immune thrombocytopenia; IVIG, intravenous immunoglobulin.

a Initial overall response was defined as an increase in platelet count to ≥30,000/μL with ≥2-fold increase from baseline after dexamethasone therapy and assessed within 30 days of the first dose of dexamethasone.

b Response types were not statistically significant after Bonferroni correction.

c Excludes 8 patients that never achieved a response. Fifteen obese patients and 26 nonobese patients were assessable.

d Alternative therapy was defined as subsequent treatment other than dexamethasone or IVIG.

e Excludes 2 patients that were lost to follow-up. Fifteen obese patients and 24 nonobese patients were assessable.

**Figure:**
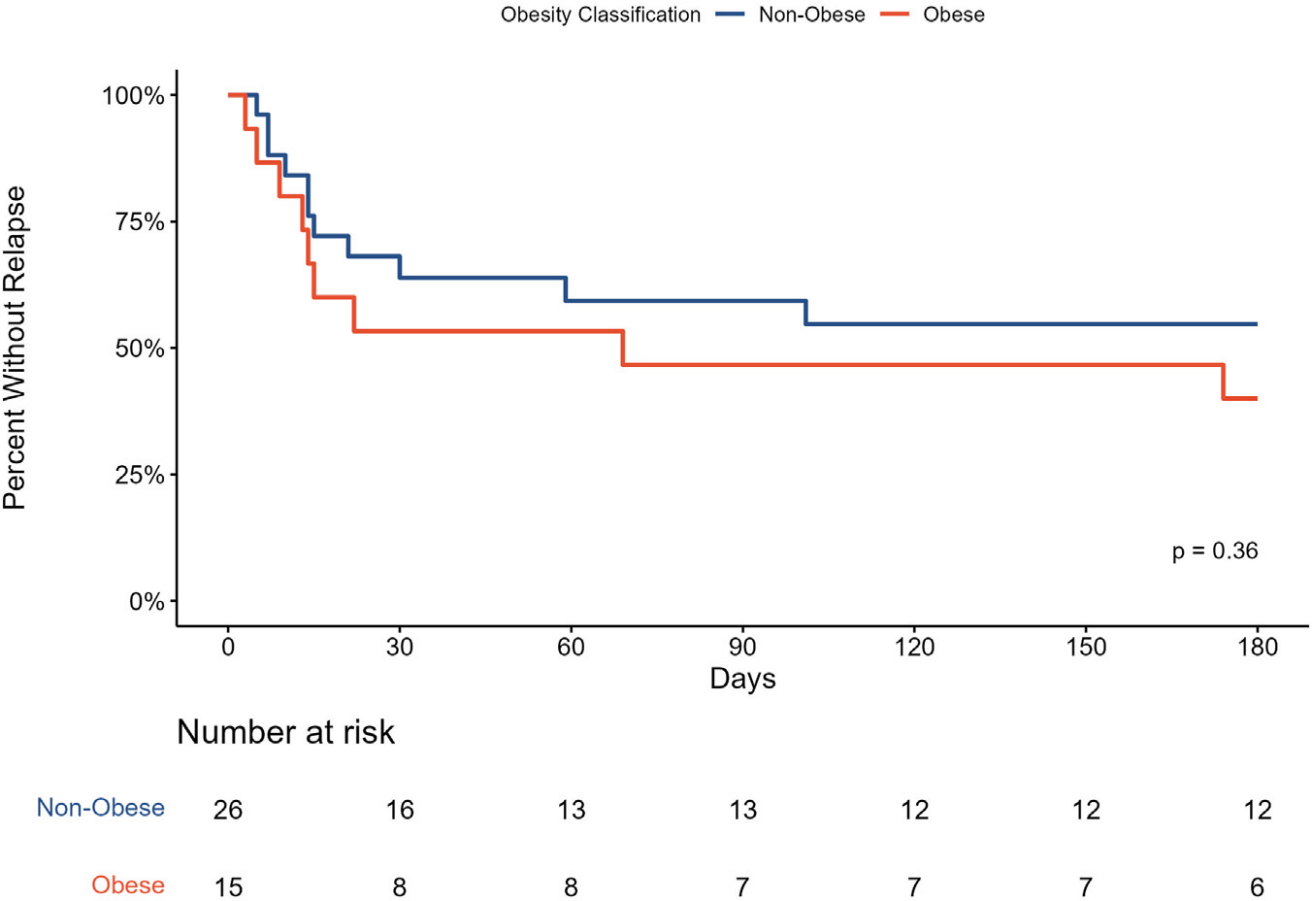
Kaplan–Meier estimates of relapse-free survival in obese vs nonobese patients.

Over 6 months of follow-up, IVIG was administered with DEX in 62% (*n* = 13) of obese patients vs 39% (*n* = 11) of nonobese patients (*P* = .12; Table 2). Seventy-one percent (*n* = 15) of obese patients and 54% (*n* = 15) of nonobese patients received alternative therapy other than IVIG (*P* = .2; Table 2). Platelet transfusion, bleeding rates, and ITP relapse were not statistically different among the groups (Table 2).

The effectiveness of DEX is well-documented when used to treat ITP, and additional DEX courses appear to be effective in those with a suboptimal initial response [[3], [4], [5], [6], [7], [8], [9], [10], [11], [12], [13]]. Our study showed patients with primary acute ITP and obesity had lower initial response rates to DEX than patients without obesity, with a nonsignificant trend favoring a higher percentage of complete remission in nonobese patients. Significant differences in secondary outcomes were not seen, perhaps due to small sample size.

To date, no prospective study has examined the effects of obesity on response to DEX for acute ITP. A single-center retrospective analysis of 275 patients with acute ITP found that overweight and obese patients were more likely to require treatment, needed more lines of therapy, and had shorter remission durations than nonobese patients [18]. Additionally, a second single-center retrospective analysis of 214 treatment-naïve patients with ITP was recently published, examining the effects of obesity on response to DEX [19]. They were able to conclude overweight and obese patients were less likely to experience an initial response than underweight or normal-weight patients (72.0% and 52.3% vs 85.7% and 85.2%; *P* = .001). This trend was also observed with complete response, sustained response, and relapse. Our study supports these findings with decreased initial responses and further expounds on these findings with a possible trend in the increased need for alternative therapies, including increased IVIG utilization.

Among the 8 patients who did not achieve a response, only 2 received a second course, while 7 received alternative, secondline therapies other than DEX, suggesting that repeat DEX courses may be underutilized. While secondline therapies play an important role in the treatment of ITP, they are often significantly more expensive while retaining the potential for serious adverse effects such as thrombosis. A recent publication reported 93% initial responses with a 41% rate of relapse or lack of response at 6 months for patients with acute ITP after DEX cycles given every 14 days × 3, results that are similar to the findings for our nonobese cohort (Table 2) [13]. Although this study did not stratify obese and nonobese patients, together with our data (Table 2), we can conclude that clinical equipoise exists when selecting the optimal number of DEX courses for all patients with ITP. Importantly, our data highlight that an initial DEX course produces variable responses between obese and nonobese patients, where nonobese are more likely to respond to a single course. Possible explanations for this remain unknown. High-dose DEX is believed to shift T-cell cytokine profile toward T helper cell 2 (Th2), restoring the Th1/Th2 imbalance often seen in ITP [20]. Restoration of this T helper cell balance has been linked to improved ITP outcomes, which may subsequently improve the response to rituximab [21]. DEX may also shift the monocyte Fc-gamma receptors (FcγR) balance toward the inhibitory FcγRIIb conformation in patients with ITP; however, the duration of action and impact of repeat DEX courses remain to be defined [22]. Taken together, it is possible that obesity contributes to a deeper perturbation of the Treg and monocyte FcγR imbalance and thus requires higher doses or additional courses to reach the prior normal homeostatic state.

Historically, clinicians have struggled with choosing secondline therapies for refractory or recurrent ITP. Currently, the American Society of Hematology guidelines for ITP do not address additional courses of DEX as a treatment option for adults with an inadequate response to 1 cycle [2]. The International Consensus Report on the Investigation and Management of Primary ITP suggests treating with 3 cycles of DEX prior to initiating secondary lines of therapy [23]. As a result, there remains substantial heterogeneity in treatment selection for this patient population and further work is needed.

Limitations of our study include enrollment at a single center and the retrospective nature of the review. Additionally, 2 nonobese patients who experienced an initial response were lost to follow-up, limiting our evaluation of relapse-free survival.

Results from this analysis suggest that despite receiving concomitant IVIG more often, obese patients with primary acute ITP have lower initial rates of response to DEX. These data argue that standard of care treatment with DEX requires further optimization, especially for obese patients with ITP. For example, for a patient weighing 100 kg, the calculated dose equivalence of 2.32 mg/kg/course derived from our nonobese patient population would equate to DEX 60 mg daily × 4 days rather than 40 mg daily for 4 days. Identification of patient populations that are more likely to exhibit refractoriness to upfront therapy has profound implications in life-threatening disease states such as ITP. Prospective, randomized studies are warranted to explore whether increased initial doses, longer courses, or repeat cycles are necessary to improve both initial response rate and duration of response to DEX in obese patients with acute ITP.
